# Ectopic acromegaly due to a GH-secreting pituitary adenoma in the sphenoid sinus: a case report and review of the literature

**DOI:** 10.1186/1756-0500-6-411

**Published:** 2013-10-12

**Authors:** Claudia Ramírez, Laura-Cristina Hernández-Ramirez, Ana-Laura Espinosa-de-los-Monteros, Juan Manuel Franco, Gerardo Guinto, Moises Mercado

**Affiliations:** 1 Endocrinology Service and Experimental Endocrinology Unit, Hospital de Especialidades Siglo XXI, Instituto Mexicano del Seguro Social, Aristoteles 68, Col. Polanco, 11560 Mexico City, Mexico; 2 Department of Neurosurgery, Instituto Mexicano del Seguro Social, Hospital de Especialidades Siglo XXI, Instituto Mexicano del Seguro Social, Mexico City, Mexico; 3 Centre for Endocrinology, William Harvey Research Institute, Barts, The University of London School of Medicine, London, UK

**Keywords:** Ectopic acromegaly, Sphenoid sinus, GH-secreting adenoma

## Abstract

**Background:**

In more than 98% of cases, acromegaly is due to a GH-secreting pituitary adenoma. The term “ectopic acromegaly” includes neuroendocrine tumors secreting GH releasing hormone (GHRH), usually located in the lungs, thymus and endocrine pancreas. Considerably less frequent are cases of ectopic acromegaly due to GH-secreting tumors located out of the pituitary fossa; except for one isolated case of a well-documented GH-secreting lymphoma, the majority of these lesions are located in the sphenoid sinus.

**Case presentation:**

We present the case of a 45 year old woman with acromegaly whose MRI showed an empty sella without evidence of a pituitary adenoma but revealed a large mass within the sphenoid sinus. She underwent transsphenoidal surgery and the excised sphenoid sinus mass, proved to be a GH-secreting adenoma; the sellar floor was intact and no other lesions were found in the pituitary fossa. She required postoperative treatment with somatostatin analogs and cabergoline for clinical and biochemical control.

**Conclusions:**

This case highlights the importance of carefully evaluating the structures surrounding the sellar area when a pituitary adenoma is not found with currently available imaging techniques. The finding of an intact sellar floor and duramater lead us to conclude that the patient’s tumor originated de novo from embryological pituitary remnants. Upon a careful review of the literature and a critical evaluation of our case we found neither clinical nor biochemical features that would distinguish an ectopic from the more common eutopically located somatotrophinoma.

## Background

Acromegaly results from an excessive GH secretion by a pituitary adenoma in over 98% of the cases [1]. It is a relatively rare condition, with a prevalence of 20–40 cases per million and an incidence of 3–4 new patients per year [1]. In less than 2% of the cases, acromegaly results from GHRH-secreting neuroendocrine tumors usually located in the lungs, thymus and endocrine pancreas [1,2]. Even less frequent are cases of real ectopic GH secretion by adenomas arising in pituitary remnants in this sphenoid sinus or by other neoplastic lesions like lymphomas [3,4].

Ectopic acromegaly due to a GHRH neuroendocrine tumor (NET) should be suspected when a patient presents with all the clinical and biochemical features of the disease but without evidence of a pituitary adenoma on MRI [2,5,6]. In this scenario, the pituitary gland may appear hyperplastic or even normal and the condition is documented by the demonstration of an elevated serum GHRH level. What follows is the localization of the NET by means of high resolution CT and ^111^In-labeled octreotide scintigraphy [2,5,6]. The absence of a pituitary adenoma in a patient with clinical and biochemical evidence of GH excess, should prompt a careful evaluation of the sphenoid sinus, since pituitary remnants, abnormally located at this site could be the source of the problem [3]. In these rare cases MRI of the region can be entirely normal or may show an empty sella [7].

We here in report the case of a woman with acromegaly without a pituitary lesion in whom GH-secreting adenomatous tissue in the sphenoid sinus proved to be the source of her hypersomatotrophism.

## Case presentation

A 45-year-old woman presented with a 5-year history of menstrual abnormalities, knee and elbow arthralgias, hands and feet enlargement, coarsening of facial features, fatigue, dental spacing and symptoms of obstructive sleep apnea (snoring and day-time sleepiness). On physical exam her blood pressure was 180/100 mmHg, pulse 80 and regular, weight 65 Kg, height 1.55 m. She had evident acral enlargement with prominence of supracilliary arches and nose bridge, prognathism and dental spacing. Numerous pigmented skin tags were visible in her anterior chest wall and there was slight acanthosis nigricans in her neck and axillary regions. Her thyroid was palpable and somewhat nodular but without distinct lesions. The diagnosis of acromegaly was confirmed by an age adjusted IGF-1 level 4.8 times the upper limit of normal (ULN) and a basal and post glucose GH concentrations of 7.7 ng/mL and 2.5 ng/mL, respectively (Table 1). The rest of anterior pituitary hormones were normal (Table 1).

**Table 1:** Preoperative hormone levels

**Hormone**	**Result**	**Normal range**
IGF-1 (ng/mL)	920	84-191
IGF-1 x ULN	4.81	< 1
Basal GH (ng/mL)	7.7	-
Nadir post-glucose GH (ng/mL)	2.5	< 0.4
Prolactin (ng/mL)	12.27	3.4-24.1
LH (mU/mL)	10.4	Postmenopausal range > 10
FSH (mU/mL)	36.4	Postmenopausal range >20
Estradiol (pg/mL)	34.7	12-211
Cortisol (μg/mL)	17.5	5-25
Free T4 (ng/dL)	1.4	0.93-1.70
TSH (mU/L)	1.95	0.27-4.20

**Figure 1:**
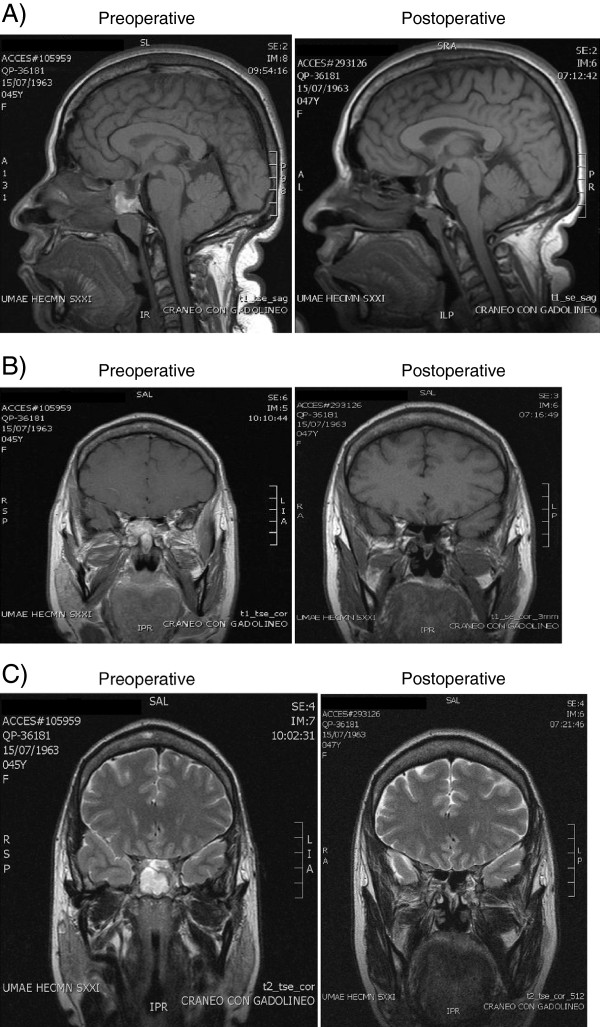
**Preoperative (left) and postoperative (right) MRI. A)** T1W sagital, gadolinium-enhanced; **B)** T1W coronal, gadolinium-enhanced; **C)** T2W coronal.

**Figure 2:**
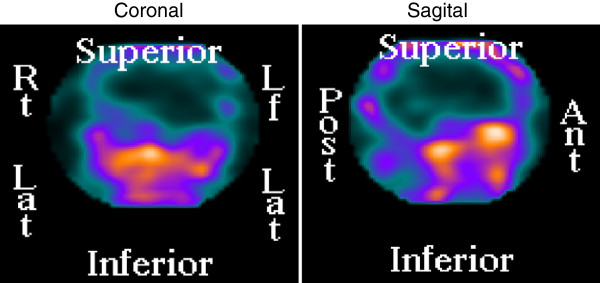
^**111**^**In-labeled octreotide scintigraphy showing abnormal uptake in the sphenoid sinus.**

MRI revealed an empty sella without clearly defined intrapituitary lesions. The sphenoid sinus was occupied by a heterogeneous but mostly hyperintense mass (Figure 1). CT scan did not reveal any abnormalities of the sellar floor. ^111^In-labeled octreotide scintigraphy showed abnormal uptake in the sphenoid sinus (Figure 2).

**Figure 3:**
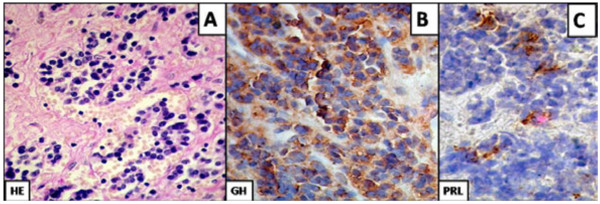
**Pathology of the resected tumor.** Panel **A** H&E shows an acidophilic adenoma. Panel **B** shows strong GH immunostaining and panel **C** reveals faint PRL immunostaining in a few cells.

Upon treatment with octreotide-LAR 20 mg monthly for 4 months, the patient reported clinical improvement of her headaches and joint pain, and her GH and IGF-1 levels decreased to 2.4 ng/mL and 3.5 times the ULN, respectively. She underwent surgical resection of the sphenoid sinus mass via an endonasal, transsphenoidal approach; the surgeon found an intact sellar floor and duramater. Both structures were opened looking for a possible intrasellar tumor, but only a protrusion of the arachnoid membrane into the sella was found. Pathological examination of the resected mass revealed an acidophilic adenoma on HE and immunohistochemistry was positive for GH (Figure 3).

**Figure 4:**
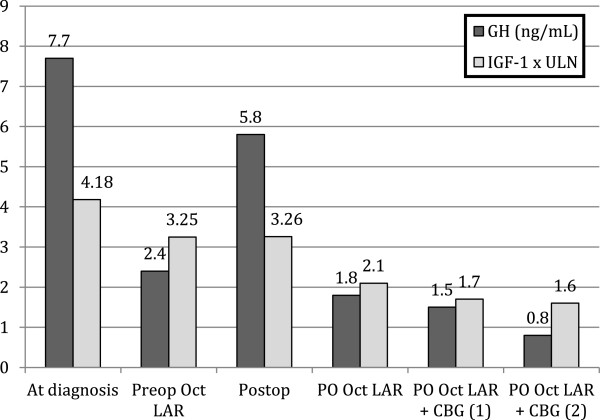
GH and IGF-1 levels at diagnosis, during primary preoperative therapy with octreotide LAR 20 mg/month, after surgery, and after postoperative therapy with octreotide LAR 20 mg/month, octreotide LAR 20 mg/month plus cabergoline (CBG) 1.5 mg/week (1), and octreotide LAR 40 mg/month plus CBG 1.5 mg/week (2).

Six months postoperatively she still had biochemically active acromegaly despite the absence of a tumor remnant on postoperative MRI (Figure 1). She was treated with a combination of octreotide LAR and cabergoline, eventually achieving an adequate hormonal control (Figure 4).

## Discussion and review of the literature

Pituitary tissue remnants can settle along the embryological migration and invagination pathway of Rathke’s pouch [8]. Such remnants have been found in the mucoperiosteum of the vomerosphenoidal articulation, the sphenoid bone, or more commonly, within the sphenoid sinus [9-11]. Since these embryological remnants lie out of the hypophyseal-portal system they are unlikely to be regulated by hypothalamic releasing hormones [12]. However, some data, albeit controversial, suggest they can respond to feedback inhibition [12]. Alternatively, the finding of adenomatous pituitary tissue within the sphenoid sinus can be explained by a pituitary tumor that protrudes inferiorly due to the pressure exerted by a large empty sella. This scenario is likely to have occurred when the sellar floor is eroded or frankly absent with the duramater extending downwards into the sphenoid sinus [3,8,10,13-16].

Ectopic acromegaly due to a GH-secreting adenoma arising from abnormally located pituitary tissue is an extremely rare entity; barely cases have been reported in the literature in the past 30 years (Table 2). The most frequent localization for these ectopic tumors has been the sphenoid sinus, followed by the clivus and isolated cases of tumors in the cavernous sinus or the suprasellar region [3,8-11,13-19]. Clinically, patients present the typical symptoms and signs of acromegaly. Sinus congestion symptoms are not commonly reported, which is interesting considering that some of the tumors fully occupy the sphenoid sinus. Biochemical documentation of acromegaly with varying combinations of basal and post-glucose GH as well as IGF-1 levels is provided in 10 of these 13 cases; PRL was measured in 7 and found to be elevated in three. No biochemical features can be found that would differentiate ectopic from eutopic acromegaly. Neither the degree of hypersomatotropinemia, nor the presence of hyperprolactinemia were associated with a particular ectopic location or the concomitant finding of an empty sella. The careful evaluation of the sphenoid sinus in our patient by MRI revealed the presence of a mass, which avidly took up ^111^In-labeled octreotide. Thus, the possibility that such mass in the sphenoid sinus was in fact the source of the excessive GH was entertained preoperatively. To our knowledge, none of the reported cases of ectopic acromegaly due to ectopically located GH-secreting adenomas have been evaluated with octreotide scintigraphy. The GH-secreting nature of the ectopic tumor was documented by immunohistochemistry in 12 of the reported cases; in six, there was concomitant, PRL immunostaining (Table 2). There is not sufficient data to ascertain if these six tumors were real mammo-somatotrophe adenomas, although that was likely the case in the three patients who had significant hyperprolactinemia. Our patient’s tumor also showed faint PRL immunostaining and yet her serum PRL was normal.

**Table 2:** Reported cases of ectopic GH-secreting adenomas

**Author**	**Bioch. Dx**	**Ectopic location**	**Pituitary**	**Dura**	**Sellar floor**	**IHC**
Corenblum, [17]	GH 46.8 ng/mL	Sphenoid sinus	Normal	Intact	Intact	GH++
	PRL 5 ng/mL					
Warner, [13]	---	Sphenoid sinus	Normal	Intact	Erosion	GH++
						PRL+
Maddona, [14]	---	Sphenoid sinus	Normal	Intact	Erosion	GH++
						TSH+
Matsuno, [15]	GH 97 ng/mL	Sphenoid sinus	Empty sella	Intact	Absent	GH++
	IGF-1 2.3 x ULN					PRL+
	PRL 140 ng/mL					α-SU+
Hori, [16]	GH 14.5 ng/mL	Sphenoid sinus	Empty sella	Defect.	Absent	GH++
	PRL 26.3 ng/mL					
Mitsuya, [9]	GH 133 ng/mL	Cavernous sinus	Normal	Intact	Intact	GH++
	PRL 73 ng/mL					PRL+
Gondim, [18]	GH 97 ng/mL	Sphenoid sinus	Empty sella	Intact	Absent	GH++
	IGF-1 2.7 x ULN					
	PRL 17 ng/mL					
Chan, [10]	---	Sphenoid sinus	Normal	Intact	Intact	---
Bhatoe, [12]	GH 36 ng/mL	Clivus	Normal	Defect.	Erosion	GH++
						PRL+
						LH+
						FSH+
Guerrero, [11]	GH 12.3 ng/mL	Sphenoid sinus	Normal	Intact	Intact	GH++
	IGF-1 1.9 x ULN					
	PRL 40.2 ng/mL					PRL+
Kurowska, [19]	GH 4.3 ng/mL	Sphenoid sinus	Empty sella	?	?	GH++
	IGF-1 2.5 x ULN					
Appel, [8]	GH 6 ng/mL	Clivus	Empty sella	Intact	Intact	GH++
	IGF-1 3.1 x ULN					PRL+
	PRL 26 ng/mL					
Hong, [3]	GH 18 ng/mL	Sphenoid sinus	Empty sella	Intact	Partially absent	GH++
	IGF-1 3.6 x ULN					
Current case	GH 7.7 ng/mL	Sphenoid sinus	Empty sella	Intact	Intact	GH++
	IGF-1 4.8 x ULN					PRL+
	PRL 12.7 ng/mL					

Among the 9 cases with lesions located in the sphenoid sinus, 5 were reported to have bone gaps or erosions of the sellar floor, and duramater defects were found in one (Table 2). Information regarding surgical outcome is described in 10 of the 13 cases found in the literature, with half of them achieving normal IGF-1 levels. In our case both, the sellar floor and the duramater were intact and although the duramater was protruding inferiorly, this could had been the result of the associated arachnoidocele. Although GH and IGF-1 levels improved after surgery in our patient, she remained biochemically active, and required combination therapy with high dose octreotide LAR and cabergoline, as it is frequently the case with eutopically located tumors. We decided against surgical reintervention in view of the absence of a tumor remnant upon follow up MRI and the low probability of achieving biochemical remission [20].

## Conclusions

In contrast to the few reported cases with this condition, our case was fully worked up preoperatively both biochemically as well as from an imaging standpoint (MRI and octreoscan scintigraphy); the excised adenomatous tissue was analyzed both by HE and immunohistochemistry for pituitary hormones, and proved to be a GH-secreting tumor. The surgical findings support the hypothesis that our patient’s adenoma originated from pituitary embryological remnants located in the sphenoid sinus. This is probably the first case of ectopic acromegaly due to a sphenoid sinus adenoma to be treated successfully with a combination of a somatostatin analog and a dopamine agonist.

## Consent

Written informed consent was obtained from the patient for publication of this Case report and any accompanying images. A copy of the written consent is available for review by the Editor of this journal.

## Abbreviations

GH: Growth hormone; IGF-1: Insulin-like growth factor type 1; LH: Luteinizing hormone; FSH: Follicle stimulating hormone; PRL: Prolactin; TSH: Thyroid stimulating hormone; FT4: Free thyroxine; GHRH: Growth hormone releasing hormone; MRI: Magnetic resonance imaging; CT: Computed tomography; ULN: Upper limit of normal; CBG: Cabrgoline.

## Competing interests

The authors have no conflict of interest to declare.

## Authors’ contributions

CR and MM drafted the manuscript. CR, LH, JMF and ALE collected all medical reports of the patient. ALE and MM were the neuroendocrinologists in charge of the case and GG was the neurosurgeon who performed the transsphenoidal resection of the tumor, contributing the technical aspects of the procedure and its findings. MM coordinated the group. All authors read and approved the final manuscript.

## Authors’ information

Claudia Ramírez, MD, MSc: Endocrinologist, Associate Professor of Medicine Universidad Nacional Autónoma de México Faculty of Medicine. Staff investigator, Experimental Endocrinology Unit, Hospital de Especialidades, CMN S.XXI, IMSS, México City. Research interests: Pituitary adenomas, Neuroendocrine tumors.

Laura-Cristina Hernández-Ramirez, MD: Endocrinologist, currently completing her PhD in molecular endocrinology at the Centre for Endocrinology, William Harvey Research Institute, Barts and The University of London School of Medicine.

Ana Laura Espinosa de los Monteros, MD: Associate Professor of Medicine Universidad Nacional Autónoma de México Faculty of Medicine. Senior attending endocrinologist, Endocrinology Service, Hospital de Especialidades, CMN S.XXI, IMSS, México City. Member of the National System of Investogators. Research interests: Pituitary adenomas, acromegaly and Cushing’s disease.

Juan Manuel Franco: Sixth year medical student, Universidad Nacional Autónoma de México Faculty of Medicine. Currently completing a 12-month research fellowship at the Experimental Endocrinology Unit, Hospital de Especialidades, CMN S.XXI, IMSS, México City.

Gerardo Guinto, MD: Professor of Neurological Surgery Universidad Nacional Autónoma de México Faculty of Medicine, Head of the Neurosurgery Service, Hospital de Especialidades, CMN S.XXI, IMSS, México City. Member of the National System of Investigators. Reviewer for Journal of Neurosurgery, Neurosurgery (editorial board member) and World Neurosurgery. Research interests: Skull base and pituitary surgery.

Moises Mercado, MD, FRCP(C): Professor of Medicine Universidad Nacional Autónoma de México Faculty of Medicine. Director Experimental Endocrinology Unit, Senior Consultant, Endocrinology Service, Hospital de Especialidades, CMN S.XXI, IMSS, México City and ABC Medical Center Neurological Unit. Member of the National System of Investigators, Editor-in-chief Revista de Endocrinologia y Nutrición. Reviewer for J Clin Endocrinology and Metab (editorial board member), Endocrinology, Pituitary, Clinical Endocrinology, Endocr Pract, Eur J Endocrinol, Arch Med Res. Research interests: Clinical, therapeutic and pathophysiological aspects of pituitary tumors, particularly acromegaly, nonfunctioning tumors and Cushing’s disease.

Justification: Ectopic Acromegaly due to GH-secreting tumors located out of the pituitary fossa is an extremely rare condition. We present a thoroughly evaluated case and performed an in-depth review of the literature in an attempt to find distinctive clinical or biochemical features that would aid in the diagnosis and treatment of these patients.

Potential reviewers: Oscar Bruno, Universidad de Buenos Aires (bodomingo@intramed.net), Ashley Grossman, Oxford University (ashley.grossman@ocdem.ox.ac.uk), Philip Chanson, Hopital Bicetre, Universite de Paris (philippe.chanson@bct.aphp.fr)
